# Intraocular Pressure Control after Trabeculectomy, Phacotrabeculectomy and Phacoemulsification in a Hispanic Population

**DOI:** 10.5005/jp-journals-10008-1164

**Published:** 2014-06-12

**Authors:** Jennifer L Jung, Cristina G Isida-Llerandi, Gabriel Lazcano-Gomez, Jeffrey R SooHoo, Malik Y Kahook

**Affiliations:** Instructor, Department of Ophthalmology, University of Colorado School of Medicine, Aurora, Colorado, USA; Glaucoma Fellowship, Asociacion para evitar la ceguera en Mexico IAP, Vicente Garcia Torres 46, San Lucas Coyoacan, DF, Maxico; Glaucoma Associate Professor, Asociacion para evitarla ceguera en Mexico IAP, Vicente Garcia Torres 46, San Lucas Coyoacan, DF, Maxico; Assistant Professor, Department of Ophthalmology, University of Colorado School of Medicine, Aurora, Colorado, USA; Professor, Department of Ophthalmology, University of Colorado School of Medicine, Aurora, Colorado, USA

**Keywords:** Intraocular pressure, Trabecutectomy, Hispanic population.

## Abstract

**Purpose:** To compare the efficacy of different surgical strategies for intraocular pressure (IOP) control in Hispanic glaucoma patients with and without visually significant cataracts.

**Design:** Comparative retrospective consecutive case series.

**Methods:** The charts of 153 consecutive patients with primary open angle glaucoma who underwent either trabeculectomy alone (n = 51), phacotrabeculectomy (n = 51), or phacoemulsification alone (n = 51) were reviewed to compare IOP control, the number of glaucoma medications required postoperatively, and the inci dence of surgical complications.

**Results:** Preoperative IOP was 17.5 ± 5.2 mm Hg in the trabe-culectomy group, 15.4 ± 4.5 mm Hg in the phacotrabeculectomy group and 13.9 ± 2.9 mm Hg in the phacoemulsification group (p < 0.001 for all comparisons). Mean IOP reduction from baseline was 4.2 ± 6.9 (24.6%) for the trabeculectomy group, 2.9 ± 5.0 (20.8%) for the phacotrabeculectomy group, and 0.9 ± 3.4 (6.5%) for the phacoemulsification group (p = 0.009). The number of IOP-lowering medications required postoperatively decreased significantly in all three groups (p = 0.001). The rate of early and late postoperative complications was similar between the trabeculectomy and phacotrabeculectomy groups and less for the phacoemulsification group.

**Conclusion:** Trabeculectomy and phacotrabeculectomy are both viable surgical options for managing open angle glau coma. Both resulted in similar rates of success, IOP reduction, decrease in use of IOP-lowering medications and post operative complication rates. Phacoemulsification alone had a lower success rate and greater need for postoperative IOP-lowering medications compared to trabeculectomy alone or phacotrabeculectomy. Phacoemulsification alone may be a reasonable option for patients with visually significant cataract and lower baseline IOP.

**How to cite this article:** Jung JL, Isida-Llerandi CG, Lazcano-Gomez G, SooHoo JR, Kahook MY. Intraocular Pressure Control after Trabeculectomy, Phacotrabeculectomy and Phaco-emulsification in a Hispanic Population. J Curr Glaucoma Pract 2014;8(2):67-74.

## INTRODUCTION

Glaucoma is a leading cause of blindness worldwide, and it is estimated that it will affect 79.6 million people by 2020.^1^ In recent years, several population-based prevalence studies have been published, reporting high rates of open angle glaucoma in the Hispanic/Latino population.^2,3^ In 2011, primary open angle glaucoma (POAG) was most commonly seen in non-Hispanic white women aged 70 to 79 in the United States.^4^ However, the largest group affected by POAG in the United States is estimated to shift to Hispanic/Latinos between 2011 and 2050 due to a number of factors specific to this population, such as high prevalence of POAG, high immigration rate, high fertility rate and relatively longer life expectancy compared to other ethnicities.^4^

The coexistence of glaucoma and cataract is commonly seen in the aging population. However, there is a lack of con sensus regarding the best surgical management for these patients. The majority of the published literature comparing the effectiveness of various surgical approaches for glaucoma studied mostly non-Hispanic white populations; the largest minority in these studies is usually black/African-American. A review of the literature suggests that cataract surgery alone can lower intraocular pressure (IOP) in patients with or without glaucoma.^5,6^ However, cataract surgery alone may not provide adequate IOP control for patients with glaucoma, and they may often need subsequent filtering surgery.^7^ In such patients, trabeculectomy can be combined with phacoemulsification. Conversely, trabeculectomy can be per formed prior to cataract surgery. This is commonly done in patients where the optic nerve is at greater risk of damage from postoperative IOP spikes after cataract surgery and the surgeon chooses to address IOP first, even with coexistent cataract. However, cataracts often progress following a trabe culectomy, which necessitates subsequent cataract removal.^8,9^ When patients undergo two sequential surgeries, they may have a longer visual rehabilitation and a higher risk of bleb failure with the second surgery.^10,11^ Some studies suggest that combined procedures may be less successful at reducing IOP than trabeculectomy alone^12^ while others report similar efficacy.^13^ Often, surgeons base their decision for a particular surgery on an individual basis, depending on many factors including the patient's IOP control, visual field progression, appearance of the optic nerve, and prior interventions. In this study, we add to this growing body of knowledge by comparing the effectiveness of IOP control and medication independence for trabeculectomy alone, combined phacotrabeculectomy, and phacoemulsification alone with a focus on a Latino population of Mexican ancestry.

## METHODS

A total of 153 patients were included in this review. This retrospective review was approved by the Institutional Review Board at Asociación para Evitar la Ceguera in Mexico (APEC) hospital. Data was collected according to The Health Insurance Portability and Accountability Act (HIPAA) privacy and security rules. There were fifty-one patients in each group: trabeculectomy alone group T, phacotrabeculectomy (group PT), and phacoemulsification alone (group P). The decision for a particular surgery was made on an individual basis depending on factors such as the patient's IOP and the number of IOP-lowering medications. Details are further explained in the results section. Patients who had undergone prior filtering surgery were excluded from the study. For group T, patients who had undergone phacoemulsification in the previous 6 months were excluded from the review. Surgeries performed by residents in training were also excluded.

For patients in group T, mitomycin-C (MMC) 0.4 mg/ml was administered intraoperatively for only five of the 51 patients as they were deemed by the surgeon to be at greater risk of fibrosis. MMC is not routinely used in the hospital where data collection took place. None of the patients in group PT received antifibrotics during surgery. Patients in group PT underwent standard two-site phacoemulsification and trabeculectomy. For patients in group C, phacoemulsification was completed through a temporal clear corneal incision and an intraocular lens (IOL) was implanted into the capsular bag. Follow-up visits were scheduled at day 1, day 3, day 5, day 7, day 15, 1, 2, 3, 4, 5 and 6 months postoperatively. Each examination included Snellen visual acuity (VA), Goldmann appla-nation tono metry, slit-lamp biomicroscopy and Seidel testing.

The primary outcome measure was the failure rates among treatment groups at 6 months postoperatively. Failure was defined as IOP greater than 21 mm Hg or less than 20% reduction below baseline on two consecutive follow-up visits after 3 months, IOP ≤ 5 mm Hg on two consecutive visits after 3 months, reoperation for glaucoma, or loss of light perception vision. Intervention requiring return to the operating room was considered as a complication. Interventions performed at the slit-lamp, including massage, needling, or suture lysis, were not counted as glaucoma reoperations or complications. Complete success was defined as eyes that had not failed by the above criteria and were not on IOP-lowering medication. Qualified success was defined as eyes that had not failed by the above criteria but required supplemental medical therapy for IOP control.

Secondary outcomes included difference in mean post operative IOP, mean IOP reduction from baseline, mean number of IOP-lowering medications, and rates of postoperative complications. These complications included IOP spike on the first postoperative day (defined as IOP > 25 mm Hg), bleb leak, hyphema, suprachoroidal detachment or hemorrhage and endophthalmitis. Hypotony was defined as IOP ≤ 5 after postoperative day 1.

### Statistical Analysis

Results are shown as the mean ± standard deviation for conti nuous variables and as frequencies and percentages for categorical variables. Snellen VA measurements were con verted to logMAR equivalents for the purpose of data analysis. Comparisons among the three treatment groups were performed using a one-way analysis of variance (ANOVA) test for continuous variables. A student's t-test was used when comparing means of two treatment groups. A Chi-squared test was performed for comparison of pro portions. Fisher's exact test was used where expected counts under the null hypothesis were less than five. Pair-wise com parisons were made using a logistic regression model with adjust ments for multiple comparisons using Tukey's honest significantly difference (HSD) test. p-values less than 0.05 were considered significant.

Analyses were performed using the R statistical package (R Core Team, 2013). Tables containing odds ratio (OR) estimates are the exponential results of the pair-wise comparison using logistic regression. Estimates less than one mean that the first group has lower odds than the second group in question. Odds ratios less than one were also presented as their reciprocal, 1/OR, to give a more easily interpreted number.

## RESULTS

### Patient Characteristics

Patient demographics are listed in Table 1. All patients had primary open angle glaucoma. All patients self-identified as Hispanic/Latino. Patients in group T were significantly younger than those in groups PT and P (p < 0.001 for both comparisons). There were more females than males in all three groups. Baseline IOP was significantly different among the three groups (group T = 17.5 ± 5.2 mm Hg, group PT = 15.4 ± 4.5 mm Hg, group p = 13.9 ± 2.9 mm Hg; p < 0.001 for all comparisons). When comparing groups T and PT alone, baseline IOP was significantly higher for group T (p = 0.0287). Patients in group P were on significantly fewer IOP-lowering medications at baseline compared to groups T and PT (p < 0.001 for both comparisons). All patients in groups T and PT were on at least one IOP-lowering medication at baseline (group T = 3.6 ± 0.9, group PT = 3.5 ± 0.9, p = 0.576). Forty-seven patients (92.2%) in group P were on medical therapy (1.4 ± 0.7).

### Intraocular Pressure Reduction

At 6 months, IOP was 13.4 ± 4.2 mm Hg for group T, 13.1 ± 3.1 mm Hg for group PT, and 12.0 ± 2.7 mm Hg for group P (p = 0.826). Mean postoperative IOP was significantly lower than baseline IOP in group T (p < 0.001) and group PT (p = 0.003), but not for group P (p = 0.111). Mean IOP reduction from baseline was 4.2 ± 6.9 (24.6%) for group T, 2.9 ± 5.0 (20.8%) for group PT and 0.9 ± 3.4 (6.5%) for group P (p = 0.009). There was no significant difference in mean IOP reduc tion between groups T and PT (p = 0.310) at 6 months postoperatively.

**Table 1:** **Table 1:** Baseline characteristics of patients

		*Group P (n = 51)* Trabeculectomy		*Group PT (n = 51)* Phacotrabeculectomy		*Group P (n = 51)* Phacoemulsification	
Age^a^ (in years)		59.7 ± 9.8 (30-80)		73.7 ± 7.5 (60-86)		74.2 ± 9.4 (56-93)	
*Sex*^b^							
Male		12 (24%)		13 (25%)		18 (35%)	
Female		39 (76%)		38 (75%)		33 (65%)	
Baseline IOP^a^ (mm Hg)		17.5 ± 5.2 (10-38)		15.4 ± 4.5 (8-32)		13.9 ± 2.95 (9-24)	
IOP-lowering medications^a^		3.6 ± 0.9 (1-5)		3.5 ± 0.9 (1-4)'		1.4 ± 0.7 (0-3)	

^a^Data presented as mean ± standard deviation (range); ^b^Data presented as number (percentage)

**Table 2:** **Table 2:** IOP and medical therapy at baseline and at 6 months follow-up

		*Group T (n = 51)* * Trabeculectomy*		*Group PT (n = 51)* *Phacotrabeculectomy*		*Group P (n = 51)* *Phacoemulsification*	
*Baseline*							
IOP (mm Hg)		17.5 ± 5.2 (10-38)		15.4 ± 4.5 (8-32)		13.9 ± 2.95 (9-24)	
Medications		3.6 ± 0.9 (1-5)		3.5 ± 0.9 (1-4)		1.4 ± 0.7 (0-3)	
*Six months visit*							
IOP (mm Hg)		13.4 ± 4.2 (6-32)		13.1 ± 3.1 (6-26)		13 ± 2.7 (9-25)	
Medications		0.37 ± 0.9 (0-4)		0.16 ± 0.7 (0-4)		0.71 ± 1.2 (0-4)	

IOP: Intraocular pressure; Data are presented as average ± standard deviation (range)

**Table 3:** **Table 3:** Estimated odds ratio of being on IOP-lowering medication at 6 months

		*Estimate*		*Lower* *(95% CI)*		*Upper* *(95% CI)*		*p-value*	
PT *vs* T		0.2563		0.0511		1.2849		0.1169	
P *vs* T		1.8743		0.6353		5.5293		0.3597	
P *vs* PT		7.3143		1.5445		34.639		0.0077	

IOP: Intraocular pressure; CI: Confidence interval; PT: Phacotra becu-lectomy group; T: Trabeculectomy group; P: Phacoemulsification group

### Medical Therapy

Table 2 shows the number of IOP-lowering medications in the three groups at baseline and at the 6 months follow-up visit. The mean number of medications was reduced in all three groups. The number of IOP-lowering medications decreased from baseline by 3.2 ± 1.1 in group T (p < 0.001), 3.4 ± 1.1 in group PT (p < 0.001), and 0.6 ± 1.2 in group P (p = 0.001). At the 6 months visit, ten patients (19.6%) in group T required IOP-lowering medication, three patients (5.9%) in group PT, and sixteen patients (31.4%) in group P (p = 0.0045). There were no significant differences in number of medications required postoperatively between groups T and PT (p = 0.117) or between groups T and P (p = 0.360). Group PT patients were on significantly fewer medications postoperatively compared to group P (p = 0.008). Table 3 shows the estimated odds ratio comparing odds of being on IOP-lowering medication after 6 months. Patients in group P have 7.3143 times the odds of those in group PT of being on IOP-lowering medications 6 months posteroperatively with a 95% confiudence interval (CI) of 1.5445 to 34.6388.

### Treatment outcomes

Treatment outcomes for the three groups are shown in Table 4. Group P had the highest failure rate (80.3%) at the end of the 6 months follow-up period, which was significantly higher compared to group T (p = 0.005) and group PT (p = 0.023). Treatment failure occurred in 24 patients (47.1%) in group T and in 26 patients (50.9%) in group PT at 6 months. Pair-wise comparison of groups T and PT showed no statistically significant difference between failure rates (p = 0.823). Patients in group P have 3.957 times the odds of those in group T of meeting criteria for failure (95% CI; 1.4322-10.93) and 3.125 times the odds of those in group PT for failure (95% CI; 1.1335-8.6158).

In group T, 25 patients (49%) were classified as complete successes and two patients (3.9%) as qualified successes. In group PT, complete success occurred in 25 patients (49%) with out any qualified success. For group P, only eight patients (15.7%) met the criteria for complete success and two patients (3.9%) for qualified successes. Pair-wise comparison of groups T and PT showed no significant difference for complete success (p = 1.00). Groups T and PT each have 5.168 times higher odds of meeting criteria for complete success compared to group P (95% CI; 0.0635-0.5895). The number of patients who met criteria for qualified success did not significantly differ among the three treatment groups (p = 0.547). Pair-wise comparisons for qualified success were not possible due to the low number of qualified successes and the lack of observation for qualified success in group PT.

Table 5 lists the reasons for treatment failure. The most common reason for treatment failure was inadequate IOP reduc tion. The number of patients with inadequate IOP reduction was significantly higher in group P compared to group T (p = 0.004) or group PT (p = 0.022). There was no significant difference between the groups T and PT (p = 0.823), One patient in group T failed due to persistent hypotony. Two patients (3.9%) in group P required additional surgeries for IOP control. They both underwent glaucoma drainage device implan tation for inadequate IOP control on maximal medical therapy. No patients in groups T or PT required reoperation for glau coma. No patient in any group had loss of light perception vision.

Of the five patients in group T who received MMC, there were three complete successes (60%) and two failures due to inadequate IOP control (40%). Even with these patients excluded from group T, there was no difference in the results. Pair-wise comparisons of groups T and PT remained in significant for complete success (p = 0.995) and for failure (p = 0.859).

**Table 4:** **Table 4:** Treatment outcomes

		*Group T (n = 51)* *Trabeculectomy*		*Group PT (n = 51)* *Phacotrabeculectomy*		*Group P (n = 51)* *Phacoemulsification*		*p-value*	
Failure^a^		23 (47.1%)		26 (50.9%)		39 (80.3%)		0.003	
Complete success^b^		25 (49%)		25 (49%)		8 (15.7%)		0.0003	
Qualified success^c^		2 (3.9%)		0		2 (3.9%)		0.5467	

^a^Failure was defined as IOP greater than 21 mm Hg or less than 20% reduction below baseline on two consecutive follow-up visits after 3 months, IOP ≤ 5 mm Hg on two consecutive visits after 3 months, reoperation for glaucoma, or loss of light perception vision; ^b^Complete success was defined as eyes that had not failed by the above criteria and were not on IOP-lowering medication; ^c^Qualified success was defined as eyes that had not failed by the above criteria but required supplemental medical therapy for IOP control

### Visual Acuity

Baseline VA was worse for groups PT and P compared to group T (p = 0.001). Final VA at 6 months was similar for all three groups (p = 0.845). VA for group T remained stable without significant changes at baseline and postoperatively (p = 0.722). VA for both groups PT and P significantly improved after cataract extraction. There was no significant difference in change in VA between groups PT and P (p = 0.206). Table 6 shows the baseline and postoperative visual acuities in Snellen and logMAR units.

### Postoperative Complications

Complications are separated into early and late postoperative categories as shown in Table 7. Early postoperative complications were defined as complications occurring in the first month after surgery. Postoperative day 1 (POD#1) IOP spike occurred at similar rates in the three groups. Early hypo-tony (IOP < 5 at equal or less than postoperative month 1) developed in sixteen patients (31.4%) in group T and in twelve patients (23.5%) in group PT (p = 0.5038). Two patients (3.9%) in group PT developed choroidal detachments. Bleb leaks occurred in five patients (9.8%) for groups T and PT. There was no significant difference in the rate of late hypotony for groups T and PT (p = 0.4337). There were no cases of early or late hypotony in group P.

## DISCUSSION

Our study evaluated the different surgical approaches for patients with coexisting POAG and cataracts, specifically in Latinos of Mexican ancestry. The results of this study suggest that both trabeculectomy and phacotrabeculectomy are equally effective for IOP control with similarly low postoperative complications. The trabeculectomy and phaco-trabeculectomy groups had similar failure rates of 47.1 and 50.9%, respectively. At 6 months, IOP reduction from baseline was 24.6% in group T and 20.9% in group PT, which were not significantly different from each other. The number of IOP-lowering medications required postoperatively was significantly reduced in both groups.

**Table 5:** **Table 5:** Reasons for treatment failure

		*Group A (n = 51)* * Trabeculectomy*		*Group B (n = 51)* * Phacotrabeculectomy*		*Group C (n = 51)* * Phacoemulsification*	
Inadequate IOP reduction^a^		23 (45.1%)		26 (50.9%)		39 (76.5%)	
Reoperation for glaucoma		0		0		2 (3.9%)	
Persistent hypotony^b^		1 (1.9%)		0		0	
Loss of light perception		0		0		0	

IOP: Intraocular pressure; Data are presented as number (percentage); ^a^IOP >21 mm Hg or not reduced by 20% below baseline on 2 consecutive follow-up visits after 3 months; ^b^IOP ≤ 5 mm Hg on 2 consecutive follow-up visits after 3 months

**Table 6:** **Table 6:** Postoperative visual acuity results at 6 months

		*Group A (n = 51)* * Trabeculectomy*		*Group B (n = 51)* * Phacotrabeculectomy*		*Group C (n = 51)* * Phacoemulsification*	
*Baseline*							
*Snellen*							
Median		20/40		20/70		20/80	
Range		20/20 ~ HM		20/20 ~ CF 1 ft		20/40 ~ HM	
*LogMAR*							
Mean ± SD		0.44 ± 0.64		0.79 ± 0.62		0.94 ± 0.81	
*Six months*							
*Snellen*							
Median		20/40		20/40		20/50	
Range		20/20 ~ HM		20/20 ~ CF 1 ft		20/20 ~ LP	
*LogMAR*							
Mean ± SD		0.48 ± 0.48		0.43 ± 0.46		0.44 ± 0.47	

SD: Standard deviation; HM: Hand motion; CF: Count fingers; LP: Light perception

**Table 7:** **Table 7:** Postoperative complication rates

		*Group A (n = 51)* *Trabeculectomy number (% )*		*Group B (n = 51)* * Phacotrabeculectomy number (% )*		*Group C (n = 51)* * Phacoemulsification number (% )*	
*Early complications (Equal or less than 1 month)*							
POD#1 IOP spike^a^		4 (7.8%)		5 (9.8%)		7 (13.7%)	
Hypotony^b^		16 (31.4%)		12 (23.5%)		0	
Bleb leak		5 (9.8%)		5 (9.8%)		N/A	
Bleb hemorrhage		1 (1.96%)		0		N/A	
Hyphema		0		0		1 (1.96%)	
Choroidal detachment		0		2 (3.92%)		0	
Retained nucleus		0		0		1 (1.96%)	
*Late complications (Greater than 1 month)*							
Hypotony^b^		2 (3.9%)		5 (9.8%)		0	

POD: Postoperative day; IOP: Intraocular pressure; Data are presented as number of patients (percentage); ^a^IOP > 25 mm Hg at postoperative day 1; ^b^IOP < 6 mm Hg after postoperative day 1

Our results are consistent with previous findings summarized in an evidence-based review by Jampel et al.^14^ They reviewed 33 articles and concluded that there was insufficient evidence to state whether either staged or combined cataract surgery and trabeculectomy was superior. Donoso and Rodriguez reported similar results for combined *vs* sequential phacotrabeculectomy with intraoperative 5-fluorouracil.^15^

Some studies have shown that trabeculectomy alone is more effective at lowering IOP.^16,17^ Friedman et al in their review of 39 articles, reported that there was weak but sufficient evidence that trabeculectomy alone lowers IOP more than combined phacotrabeculectomy.^12^ Kleinmann et al reviewed the charts of 135 patients who have undergone phacotrabeculectomy or trabeculectomy alone and found that IOP reduction was significantly larger for trabeculectomy alone. However, they noted that the trabeculectomy group had a significantly higher preoperative IOP compared to the combined group, which can predispose them to a greater IOP drop with surgery.^16^ Murthy et al, in their study of 190 eyes that underwent trabeculectomy or phacotrabeculectomy (both with MMC), reported a significantly greater mean IOP reduction with the trabeculectomy group but attributed this finding to the significantly higher baseline IOP for trabecu-lectomy group compared to the combined group. Thus, they concluded that both surgeries had comparable safety profiles and similar efficacy after 2 years of follow-up.^13^

In our study, patients who underwent phacoemul sigification alone had significantly less IOP reduction and also required more IOP-lowering medications compared to the other two groups. This is consistent with the review by Friedman et al which stated that IOP reduction is lower for phacoemulsification compared to a combined procedure.^12^ They reported that phacoemulsification can result in an average of 2 to 4 mm Hg reduction in IOP in patients with glaucoma whereas phacotrabeculectomy can lower IOP by approximately 8 mm Hg for a mean of 1 to 2 years.^12^ Casson et al stated that cataract surgery can produce IOP reduction, but the effect is generally small (1 to 4 mm Hg).^18^ The degree of IOP reduction after cataract surgery may also be a function of preoperative IOP in both normotensive and ocular hypertensive eyes.^19,20^ Mathalone reported that although IOP reduction may occur postoperatively, IOP may return to baseline with time. In the ocular hypertension treatment study (OHTS), Mansberger showed that there was a 14.5% decrease in IOP after cataract surgery which was sustained for 1 year, but the effect diminished over the subsequent 2 years.^19^ A similar report was published by Suzuki et al in their study of IOP after phacoemulsification in patients without glaucoma, where they found that there was no significant change between the mean preoperative and postoperative IOP after 10 years.^21^

Success of treatment was subdivided into complete and qualified successes, depending on the need for supplemental medical therapy. Group P had lower rates of both complete and qualified successes compared to groups T and PT. There was no significant difference between the success rates for groups T and PT. However, all three groups had a significant reduc tion in the number of IOP-lowering medications post-operatively compared to baseline, suggesting that even phacoemulsification alone is a viable option for patients who desire to become more medication independent.

The similarly low rates of early and late postoperative complications between groups T and PT provides further evidence that trabeculectomy and phacotrabeculectomy may be equally safe. All three groups had similarly low rates of POD #1 IOP spikes. Bleb leaks and hypotony occurred at similarly low rates for both groups T and PT. There were no cases of suprachoroidal hemorrhage or endophthalmitis in any group.

Owing to the lack of randomization in our study, several baseline differences among the three treatment groups could have contributed to the differences in the results. Pre-operative IOP was significantly different among the three groups, which can affect the degree of postoperative IOP reduction from baseline. As previously mentioned, various studies have reported a linear relationship of pre operative and postoperative IOP after trabeculectomy or phaco-emulsification, with higher preoperative IOP leading to greater IOP reduction.^13,20,22^ Watson and Grierson found that a mean reduction of 10.3 mm Hg in patients with preoperative IOP of 25 mm Hg increased to 24.7 mm Hg with preoperative IOP of 40 mm Hg after trabeculectomy.^22^ Phaco emulsification group (group P) had a significantly lower preoperative IOP compared to groups T and PT. This difference is closely related to the different indications for surgery since patients in group P had lower baseline IOP and were noted in their charts to be well-controlled on medical therapy compared to the other groups and did not require glaucoma surgery at the time of phacoemulsification. The lower preoperative IOP may contribute to the observed lower IOP reduction (6.5%) and higher failure rate (80.3%) for group P. The mean postoperative IOP was not significantly lower than preoperative IOP for group P, but significantly fewer patients required IOP-lowering medication to reach target IOP. This suggests that although phacoemulsification alone may not lead to adequate IOP reduction, it can still be beneficial for patients who desire to be on fewer medications postoperatively to meet target IOP.

Patients in group T were significantly younger than patients in groups PT and P. Preoperative visual acuities were significantly worse for groups PT and P compared to group T, attributed to visually significant cataracts in groups PT and P, which led to the decision to perform cataract extraction. These differences also refect the different circum stances and indications for surgery in the three groups. Patients in group T were generally younger, and most did not have visually significant cataracts, which explains the better visual acuities preoperatively. Postoperative visual acuities were similar among the three groups at the 6 months follow-up visit. Trabecu lectomy has been shown to significantly increase the incidence of cataract progression,^8,9,23^ but in our study, post operative visual acuity for group T did not significantly worsen at 6 months. This can be explained by the relatively short follow-up interval in our study since cataract progression may occur overa longer time period. Adelman et al reported that in young patients (average age 43.7) who underwent trabeculectomy, 24% of patients required cataract surgery at mean of 26 months after filtering surgery.^23^

Although the trabeculectomy group had a significantly higher baseline IOP compared to the phacotrabeculectomy group, the mean IOP reduction was similar in the two groups. With the higher baseline IOP, we would have expected to see a larger IOP reduction, based on the findings that were stated previously. In addition, more patients required IOP-lowering medications in group T than in group PT although the mean medication reduction was not statistically different between the two groups. This observation may be due to the more aggressive nature of POAG in patients in group T compared to group PT. Patients in group T were significantly younger than those in groups PT or P but still had poorly controlled IOP on maximal medical therapy.

It is a widely accepted fact that black race is a strong risk factor for failure for trabeculectomy alone ^24^ or for combined phacotrabeculectomies.^25,26^ A more rapid wound healing process has been hypothesized as an explanation for this finding.^27^ The relative paucity of studies on Latino patients makes it difficult to ascertain, but Hispanic patients, similar to black patients, may also have a more aggressive wound healing process that increases their risk for failure of filtering surgeries. Epidemiologic studies on keloids have shown that darker-skinned patients are 15 times more likely to develop an abnormal fibroproliferating healing process leading to keloids compared to whites.^28^ The incidence of keloids is reported to be as high as 16% in both black and Hispanic population.^28^ Although the pathogenesis of scar formation is different for post-filtering surgery fibrosis and keloid formation, the aggressive and rapid wound healing occurring in black patients may also be present in Hispanic/Latino patients. More studies are necessary to specifically delineate whether Hispanic/Latino ethnicity is an independent risk factor for increased filtering surgery failure.

Only five patients received intraoperative antifibrotic therapy MMC as our data was collected in an institution where MMC is not routinely used. These patients received MMC due to certain characteristics that increased their risk for failure, such as determined by the surgeon. Thus, our results may not be generalizable to other areas with different practice patterns. Cohen et al reported the benefit of MMC in their placebo-controlled, double-masked evaluation of MMC in combined glaucoma and cataract procedures.^29^ The mean IOP reduction was significantly greater in the MMC group compared to a placebo group although there were more postoperative complications in the MMC group. The MMC group was on fewer medications postoperatively but when adjusted for baseline difference in medication use, it was not statistically significant.^29^ The high failure rates in our study may be in part due to the lack of antifibrotic use in the majority of our patients. Conversely, Budenz et al retrospectively reviewed patients who underwent phacotrabeculectomy with MMC, 5-FU, or without antifibrotics. The MMC group had lower IOP compared to 5-FU group, but not when compared to the group without antifibrotics. They concluded that phacotrabeculectomy is a successful surgery with or without antifibrotic use.^25^ It is difficult to draw conclusions as only a small number of patients received MMC. However, even with those five patients excluded from the data set, there was no diffe rence in the results of the study.

There are several limitations to our study, especially the retrospective nature of the study. The lack of randomization for treatment groups may have resulted in selection bias due to differences in baseline characteristics as described previously, different indications for surgery and varied target IOP. However, the intention of this study was to investigate the natural history of IOP outcomes and surgical results with the three various approaches studied and this was accomplished. Comparisons between groups are instructive but must be viewed with the understanding that the demographics and clinical situations were not uniform. Our relatively short follow-up period also limits our study, since long-term IOP control and success rates are unknown. We also understand that treatment success or failure cannot be defined by an arbitrarily set IOP level and that true treatment success should be measured by prevention of further optic nerve damage. However, since IOP lowering is still the main goal of glaucoma therapy at this time, we utilized the same outcome criteria that were prospectively defined in the tube *vs* trabeculectomy (TVT) study.^30^

Overall, our study demonstrates that trabeculectomy and two-site phacotrabeculectomy are similar in efficacy and safety for surgical management of glaucoma. Phacoemulsi-fication alone had less IOP reduction and greater use of medications postoperatively compared to the trabeculectomy and phacotrabeculectomy groups, but target IOP was achievable with fewer medications than preoperatively. This suggests that phacoemulsification alone may be a viable option for patients who have a higher target IOP or those who are willing to remain on medications postoperatively.

## References

[1] Quigley HA, Broman AT (2006). The number of people with glaucoma worldwide in 2010 and 2020. Br J Ophthalmol.

[2] Quigley HA, West SK, Rodriguez J, Munoz B, Klein R, Snyder R (2001). The prevalence of glaucoma in a population-based study of Hispanic subjects: Proyecto VER.. Archi Ophthalmol.

[3] Varma R, Ying-Lai M, Francis BA, Nguyen BB, Deneen J, Wilson MR, Azen SP (2004). Prevalence of open-angle glaucoma and ocular hypertension in Latinos: the Los Angeles Latino Eye Study.. Ophthalmology.

[4] Vajaranant TS, Wu S, Torres M, Varma R (2012). The changing face of primary open-angle glaucoma in the United States: demographic and geographic changes from 2011 to 2050. Am J Ophthalmol.

[5] Samuelson TW, Katz LJ, Wells JM, Duh YJ, Giamporcaro JE (2011). Randomized evaluation of the trabecular micro-bypass stent with phacoemulsification in patients with glaucoma and cataract.. Ophthalmology.

[6] Shingleton BJ, Gamell LS, O' Donoghue MW, Baylus SL, King R (1999). Long-term changes in intraocular pressure after clear corneal phacoemulsification: normal patients versus glaucoma suspect and glaucoma patients.. J Cataract Refract Surg.

[7] Mathalone N, Hyams M, Neiman S, Buckman G, Hod Y, Geyer O (2005). Long-term intraocular pressure control after clear corneal phacoemulsification in glaucoma patients.. J Cataract Refract Surg.

[8] (2001). AGIS (Advanced Glaucoma Intervention Study) Investigators. The Advanced Glaucoma Intervention Study: 8. Risk of cataract formation after trabeculectomy.. Arch Ophthalmol.

[9] Lichter PR, Musch DC, Gillespie BW, Guire KE, Janz NK, Wren PA, Mills RP (2001). Interim clinical outcomes in the Collaborative Initial Glaucoma Treatment Study comparing initial treatment randomized to medications or surgery.. Ophthalmology.

[10] Rebolleda G, Muñoz-Negrete FJ (2002). Phacoemulsification in eyes with functioning filtering blebs: a prospective study.. Ophthalmology.

[11] Yamagami S, Araie M, Mori M, Mishima K (1994). Posterior chamber intraocular lens implantation in filtered or nonfiltered glaucoma eyes.. Jpn J Ophthalmol.

[12] Friedman DS, Jampel HD, Lubomski LH, Kempen JH, Quigley H, Congdon N, Levkovitch-Verbin H, Robinson KA, Bass EB (2002). Surgical strategies for coexisting glaucoma and cataract: an evidence-based update.. Ophthalmology.

[13] Murthy SK, Damji KF, Pan Y, Hodge WG (2006). Trabeculectomy and phacotrabeculectomy, with mitomycin-C, show similar two-year target IOP outcomes.. Can J Ophthalmol.

[14] Jampel HD, Lubomski LH, Friedman DS, Robinson KA, Congdon N, Quigley HA, Levkovitch-Verbin H, Kempen J, Bass EB (2001). Surgical treatment of coexisting cataract and glaucoma.. Evid Rep Technol Assess (Summ).

[15] Donoso R, Rodriguez A (2000). Combined versus sequential phacotrab-eculectomy with intraoperative 5-fluorouracil.. J Cataract Refract Surg.

[16] Kleinmann G, Katz H, Pollack A, Schechtman E, Rachmiel R, Zalish M Comparison of trabeculectomy with mitomycin C.

[17] Lochhead J, Casson RJ, Salmon JF (2003). Long term effect on intraocular pressure of phacotrabeculectomy compared to trabe culectomy.. Br J Ophthalmol.

[18] Casson RJ, Salmon JF (2001). Combined surgery in the treatment of patients with cataract and primary open-angle glaucoma.. J Cataract Refract Surg.

[19] Mansberger SL, Gordon MO, Jampel H (2012). Reduction in intraocular pressure after cataract extraction: the Ocular Hypertension Treatment Study.. Ophthalmology.

[20] Poley BJ, Lindstrom RL, Samuelson TW (2008). Long-term effects of phacoemulsification with intraocular lens implantation in normotensive and ocular hypertensive eyes.. J Cataract Refract Surg.

[21] Suzuki R, Kuroki S, Fujiwara N (1997). Ten-year follow-up of intraocular pressure after phacoemulsification and aspiration with intraocular lens implantation performed by the same surgeon.. Ophthalmologica.

[22] Watson PG, Grierson I (1981). The place of trabeculectomy in the treatment of glaucoma.. Ophthalmology.

[23] Adelman RA, Brauner SC, Afshari NA, Grosskreutz CL (2003). Cataract formation after initial trabeculectomy in young patients.. Ophthalmology.

[24] Miller RD, Barber JC (1981). Trabeculectomy in black patients.. Ophthalmic Surg.

[25] Budenz DL, Pyfer M, Singh K, Gordon J, Piltz-Seymour J, Keates EU (1999). Comparison of phacotrabeculectomy with 5-fluorouracil, mitomycin-C and without antifibrotic agents.. Ophthalmic Surg Lasers.

[26] Shin DH, Hughes BA, Song MS, Kim C, Yang KJ, Shah MI, Juzych MS, Obertynski T (1996). Primary glaucoma triple procedure with or without adjunctive mitomycin. Prognostic factors for filtration failure.. Ophthalmology.

[27] Broadway DC, Chang L P (2001). Trabeculectomy, risk factors for failure and the preoperative state of the conjunctiva.. J Glaucoma.

[28] Chike-Obi CJ, Cole PD, Brissett AE (2009). Keloids: pathogenesis, clinical features and management.. Semin Plast Surg.

[29] Cohen JS, Greff LJ, Novack GD, Wind BE (1996). A placebo-controlled, double-masked evaluation of mitomycin C in combined glaucoma and cataract procedures.. Ophthalmology.

[30] Gedde SJ, Schiffman JC, Feuer WJ, Herndon LW, Brandt JD, Budenz DL (2007). Treatment outcomes in the tube versus trabe culectomy study after one year of follow-up.. Am J Ophthalmol.

